# Cinnamaldehyde Attenuates the Expression of IBA1 and GFAP to Inhibit Glial Cell Activation and Inflammation in the MPTP-Induced Acute Parkinson's Disease Model

**DOI:** 10.1155/padi/9973140

**Published:** 2024-12-24

**Authors:** Panpan Jiao, Yingfeng An, Suhui Wu, Hanbing Li, Genlin Li

**Affiliations:** School of Medicine, Henan University of Chinese Medicine, Zhengzhou, China

**Keywords:** cinnamaldehyde, GDNF, GFAP, IBA1, NF-*κ*B p65, Parkinson's disease

## Abstract

Cinnamaldehyde (CA), the primary bioactive compound in cinnamon (*Cinnamomum cassia* Presl, Lauraceae, *Cinnamomum*), holds potential therapeutic benefits for Parkinson's disease (PD). To scrutinize the impact and mechanisms of CA on 1-methyl-4-phenyl-1,2,3,6-tetrahydropyridine (MPTP)-induced PD, male C57BL/6 mice were randomly allocated to CA (150, 300, and 600 mg/kg), model, Madopar, and control group (*n* = 12). The Open Field, Pole-jump, and Rotarod experiments assessed exercise capacity and anxiety levels. HPLC evaluated the levels of neurotransmitters. Immunohistochemistry was utilized to detect the expression of TH and GFAP. WB and RT-qPCR determine the expression levels of apoptosis-related genes and proteins in the substantia nigra and striatum. The findings revealed that CA not only enhanced motor abilities and reduced anxiety but also elevated the levels of TH, DOPAC, DA, 5-HIAA, HVA, and 5-HT in the substantia nigra and striatum. Moreover, it protected DA neurons and downregulated the expression of *Bax*, *Casp3*, and *Bax/Bcl-2* mRNA and proteins, while increasing the expression of *Bcl-2* mRNA compared to the model group. Furthermore, CA was observed to inhibit glial cell activation, leading to reduced levels of GFAP and IBA1 in the substantia nigra and striatum. This resulted in decreased expression of inflammatory factors such as iNOS and NF-*κ*Bp65 proteins in these regions, consequently mitigating neuroinflammation. These results suggest that CA exerts a neuroprotective effect in acute PD model mice by suppressing glial cell activation, modulating the expression of apoptotic genes, and alleviating neuroinflammation and apoptosis induced by MPTP.

## 1. Introduction

Parkinson's disease (PD) constitutes a chronic and progressive neurological disorder that impacts the central nervous system and leads to the degeneration of specific neurons in the brain [1]. A hallmark of PD is a notable reduction in dopaminergic (DA) neurons within the Substantia Nigra compacta (SNpc), resulting in impairment of the SN-striatum pathway [2]. Epidemiological data indicates that over 6 million individuals worldwide grapple with PD, and the incidence in those aged over 60 can reach 1% [3, 4]. Presently, drug therapy stands as the primary clinical intervention for PD, frequently complemented by deep brain stimulation (DBS) and rehabilitation therapy [5].

While notable progress has been made in ameliorating motor symptoms in PD, the fundamental issue of neurodegeneration remains unaddressed. The management of neurodegenerative conditions, such as PD, holds a pivotal position in neuroscience research.

The etiology of PD involves a complex interplay of genetic, environmental, and age-related factors. Ongoing research suggests that PD's onset may stem from the intricate interaction of genetic and environmental elements. Due to the intricacies of the disease, the pathophysiological mechanism of PD remains elusive. Current research predominantly centers on neuroinflammation, apoptosis, oxidative stress, α-Syn aggregation, and mitochondrial dysfunction.

The 1-methyl-4-phenyl-1,2,3,6-tetrahydropyridine (MPTP) model proves to be a valuable tool for replicating the pathophysiological and behavioral alterations seen in PD. Widely utilized across various genera, MPTP, a neurotoxin, induces PD-like symptoms by damaging DA neurons in the SN. MPTP itself, nontoxic, traverses the blood–brain barrier and undergoes metabolization into the toxic 1-methyl-4-phenylpyridinium (MPP^+^) by glial cell–produced MAO-B in the brain. MPP^+^ inhibits mitochondrial complex I, reduces ATP synthesis, and elevates free radical production, ultimately leading to degeneration and demise of DA neurons.

Cinnamaldehyde (CA), the principal active component of *Cinnamomum cassia* Presl, possesses antioxidant [6], anti-inflammatory [7], antibacterial [8], antitumor [9], antidiabetic [10], lipid-lowering [11], and other effects. Reports suggest that CA exhibits neuroprotective and memory-enhancing characteristics, capable of delaying or inhibiting neurodegenerative diseases [12]. However, few studies have been explored on whether CA can exert neuroprotective effects in the MPTP mouse model. This study aimed to establish an animal model of acute PD by MPTP, to investigate the neuroprotective effects and mechanisms of CA, and to provide new insights and methods for the prevention and treatment of PD.

## 2. Material and Methods

### 2.1. Animals and Experimental Protocols

A total of 72 male C57BL/6 mice (8–10 weeks, 23.0 ± 1.0 g, SCXK [Shandong] 20150009, Jinan Pengyue Experimental Animal Breeding Co., Ltd.) were maintained on standard chow and tap water at 22°C ± 2°C, following a 12-h light–dark cycle in a sterile animal laboratory for 18 days. After 3 days of acclimatization, the mice were randomly assigned into 6 groups according to their body weight using a random number table: Control, Model, Madopar, and CA-150, 300, and 600 mg/kg groups, each group has 12 samples.

**Figure 1:**
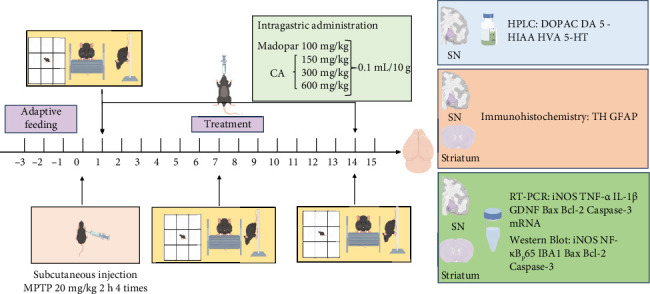
Schematic diagram of the experimental design.

The acute PD model was induced using the experimental protocol of St-Amour I [13], where MPTP (20 mg/kg, 0.9% saline, China, Shanghai Yuanye Biotechnology Co., Ltd., S31504) was intraperitoneally administered four times every 2 h. The control group received an equivalent volume of normal saline. After 24 h, various concentrations of CA solution (China, Nanjing Xinhou Biotechnology Co., Ltd.; xH50190812), 0.15, 0.3, and 0.6 g of CA were accurately weighed and dissolved in 0.5% CMC-Na solution (CMC-Na: Purified water = 0.5 g: 100 mL) and then mixed by ultrasonication in a 10 mL volumetric flask. The CA group was given different concentrations of CA solution, the positive drug group was given Madopar solution (0.9% saline: Madopar = 10 mL: 0.1 g), and the control and model groups were injected with an equal amount of 0.9% saline once a day for 14 consecutive days. The experimental design is shown in Figure 1. After the last MPTP injection (before the first gavage treatment), the mice were subjected to open field tests, rotarod tests, and pole-jump experiments to examine the behavioral science of the mice.

### 2.2. Laboratory Apparatus

ZB-200 Fatigue Rotary Rod Instrument (China, Chengdu Taimeng Software Co., Ltd.); HERAEUS FRESCO 17 high-speed refrigerated centrifuge (USA, Thermo Fisher); easy WeLL ultrapure water meter (China, Shanghai Rongyan Instrument Co., Ltd.); MP6001 electronic balance (USA, Denver Instrument Co., Ltd.); electrophoresis instrument (USA, BIO-RAD company); universal gel imaging analysis system (BIO-USA, RAD ChemiDoc MP); real-time fluorescent quantitative PCR instrument (USA, Thermo Scientific).

### 2.3. Related Behavioral Observation

#### 2.3.1. Open Field Test [14]

On the 1st, 7th, and 14th days of the experiment, each animal underwent assessment in an open-field apparatus to gauge exercise levels, exploratory behavior, and anxiety-like tendencies. The test arena, measuring 50 × 50 × 50 cm, was divided into nine squares. Parameters recorded within 5 min included the number of crossings, instances of standing on the hind limbs, and occurrences of urine and stool for each mouse. Following completion, each mouse underwent gentle cleansing with 75% ethanol to eliminate potential odors that could compromise experimental accuracy.

#### 2.3.2. Pole-Jump Test [15]

A custom-made rod, measuring 1 cm in diameter and 55 cm in height, featuring a 2-cm-diameter rubber ball at its summit, was affixed vertically to a metal base. The metal rod was enveloped in gauze to prevent mouse slippage. Mice were allowed to traverse the rod naturally until reaching the platform with their double forelimbs. The total duration mice took to traverse from the rod's apex to the platform was recorded. Each trial, separated by a minimum of 10 min, was conducted following a 3-day training period for all mice. If mice halted or reversed during the test, measurements were repeated.

#### 2.3.3. Rotarod Test [16]

The rotarod treadmill, comprising a 2.5 cm diameter bar, was sectioned into five compartments by four partitions, allowing simultaneous ambulation for five mice. Before the formal experiment, the mice underwent a 5-min pretraining session at 10 revolutions per minute (r/min) for three consecutive days. The accelerating rotor mode (from 5 to 40 r/min) was employed in the experiment. The mice were positioned on the rod with their backs facing the direction of rotation. The timer commenced at 5 r/min, recording the time when the animal descended from the rod. Each trial was iterated three times with intervals exceeding 1 h, and the average duration from the three trials was calculated.

### 2.4. Immunohistochemistry Test

Brain tissue sections were incubated overnight at 4°C with the TH primary antibody (USA, Bioworld, BS1369) and GFAP primary antibody (China, Protein Technology, 75,494). Subsequently, the sections were subjected to three washes with PBS and incubated with a secondary antibody for 50 min in the dark. Following another three washes with PBS, the sections were treated with a DAB color solution. Control the display time and immerse the sections in tap water to arrest the color process. Image-Pro Plus 6.0 software facilitated statistical analysis to determine the average optical density of TH and GFAP-positive staining in the SN and striatum sections of the midbrain.

### 2.5. High-Performance Liquid Chromatography (HPLC)

Tissue samples were rinsed and dried to remove surface moisture. After weighing, a 0.4 mol/L perchloric acid homogenate, precooled at 4°C, was added at a ratio of 1 g:10 mL and centrifuged at a low temperature (14,000 r/min, 15 min, 4°C). The supernatant was filtered through a 0.22-μm filter membrane. The chromatographic column employed was C18 (2.1 × 100 mm, 3 μm), with the mobile phase A consisting of sodium dihydrogen phosphate (100 mmol/L), sodium citrate (50 mmol/L), EDTA (50 μmol/L), sodium octane sulfonate (1.7 mmol/L), and potassium chloride (2 mmol/L). The mobile phase B was methanol, with a ratio of A:B = 95:5. The flow rate of the mobile phase was set at 0.3 mL/min, and the column temperature was maintained at 31°C. The electrochemical detector utilized a glassy carbon working electrode, with a working voltage of 520 mV, a gain of 100 nV, and an injection temperature of 4°C. Dihydroxyphenylacetic acid (DOPAC), dopamine (DA), 5-hydroxy indole acetic acid (5-HIAA), homovanillic acid (HVA), and 5-hydroxytryptamine (5-HT) were detected with an injection volume of 20 μL.

### 2.6. Real-Time PCR

The mRNA levels of *iNOS*, *TNF-α*, *IL-1β*, *GDNF*, *BAX*, *Bcl-2*, and *Casp3* were determined via real-time PCR. After RNA extraction from brain tissues, a NanoDrop 2000 (USA, Thermo Scientific) spectrophotometer was used to determine the concentration of RNA in each group, which was reverse transcribed to cDNA using the Takara (PrimeScriptTMRT reagent Kit) kit. Then, the reaction solution was configured according to the Takara (TBGreenTM Premix Ex TaqTM II) kit. The reaction was performed using QuantStudio 7 Flex RT-qPCR (USA, Thermo Scientific) instrument. The reaction conditions were as follows: using SYBR green I masterbatch, 40 cycles of amplification, premetamorphosis (95°C, 10 min), 40 cycles (95°C, 15 s ⟶ 60°C, 60 s), and melting curve (60°C ⟶ 95°C, warming up by 0.3°C every 15 s) were carried out, respectively. The fluorescence signal acquisition point was set to real-time detection at the end of each extension temperature cycle (60°C). Target gene expression was calculated using the 2^−ΔΔCt^ method. The primer sequences are presented in Table 1.

### 2.7. Western Blot

Brain tissue (20 mg) was homogenized and then centrifuged (4°C, 14,000 r/min, 5 min), with the resultant supernatant collected. Each well received 20 μg of protein, and its concentration was determined using a BCA kit (Nanjing, China, Batch No.: 20191022). Following the addition of SDS-PAGE protein loading buffer (5X), the protein underwent denaturation through heating at 100°C. Gel electrophoresis proceeded for approximately 2.5 h (concentrated gel at 70 V, separation gel at 110 V), succeeded by electrotransformation (constant current at 200 mA, 1 h), with the system then left to close at room temperature for 2 h.

Rabbit anti-iNOS, NF-*κ*B p65, Bax, Bcl-2, and caspase-3 (China, protein batch numbers: 91032, 91781, 91781, 91702, 91801) were diluted with 5% BSA/TBST (ratios of 1:2000, 1:2000, 1:1000, 1:2000, and 1:1000, respectively). IBA1 (United States, Affinity Biosciences, lot number: DF7217, 1:1000) and β-actin (Bioworld, USA, lot number: AP0060, 1:10,000) antibodies were incubated at 4°C, washed with TBST (10 min, 3 times), and subsequently diluted with HRP-labeled goat anti-rabbit IgG (1:2000). The incubation continued at room temperature for 1 h, followed by washing with TBST (10 min, 3 times), detection, observation with ECL chemiluminescence solution, and analysis using Image Lab image analysis software.

### 2.8. Data Analyses

SPSS 25.0 statistical software was used for data analysis, and the results were expressed as $\overset{¯}{x}\pm s$ for body mass, RT-PCR, and WB data. GraphPad Prism 9.5 plotting software was used to plot the results, represented as scatterplots and box plots of “median and 5–95 percent.” We used one-way ANOVA and multiple comparisons to assess the effects of CA treatment on body weight, behavior, neurotransmitters, and related genes and proteins in mice. When the variances were equal, multiple comparisons were made using the LSD method. Dunnett's T3 test was used when variances were not equal. *p* < 0.01 or *p* < 0.05 was considered a very statistically significant or significant difference between groups.

## 3. Results

### 3.1. Recovery of Body Weight in MPTP-Induced PD Model Mice by CA

**Figure 2:**
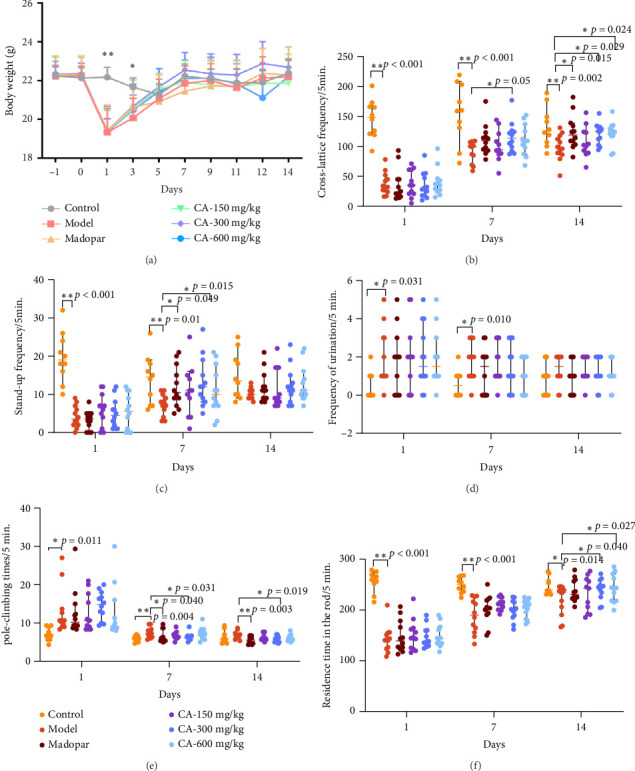
Impact of CA on body weight and exercise capacity in PD model mice (*n* = 10). (a) Changes in body weight during the experiment; (b–d) alterations in cross-lattice frequency/5 min, standing-up frequency/5 min, and urination frequency/5 min during different periods in the open-field experiment; (e) time required for mice in each group to climb the pole at various intervals in the pole climbing experiment; (f) duration each group's mice remained on the rotating rod in the rotating rod experiment. ⁣^∗^*p* < 0.05, ⁣^∗∗^*p* < 0.01 compared to the model group.

The model mice exhibited considerable weight loss compared to the control group throughout the experimental period (*p* < 0.05). Following administration, the body weight of mice in the CA group increased without significant differences (*p* > 0.05) (Figure 2(a)).

### 3.2. Enhancement of Athletic Ability by CA

In the open-field test, mice in the CA-150, 300, and 600 mg/kg groups demonstrated a noteworthy increase in the number of crossings and standing times on the 7th (*p* < 0.05) and 14th (*p* < 0.05) days compared to the model group. The CA-300 mg/kg dosage group displayed optimal performance. Although the frequency of urination decreased in the CA-150, 300, and 600 mg/kg groups, the difference was not statistically significant (*p* > 0.05). The CA-150, 300, and 600 mg/kg groups showed significantly reduced total pole-climbing time on days 7 and 14 (*p* < 0.05) in the pole-climbing experiment. Additionally, the CA-300 and 600 mg/kg groups showed significantly prolonged residence duration time of mice on the 14th day (*p* < 0.05) in the fatigue rotarod test (Figures 2(b)–2(f)).

### 3.3. Increase in TH Content, Neuronal Protection, and Apoptosis Reduction by CA

**Figure 3:**
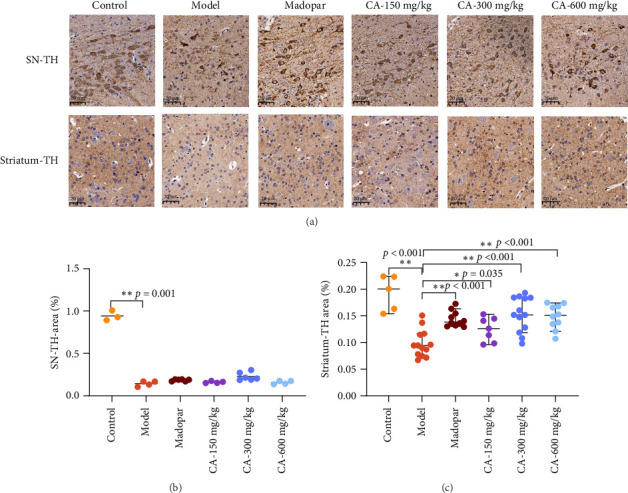
Influence of CA on the average optical density of tyrosine hydroxylase (TH) in the substantia nigra (SN) and striatum of MPTP-induced acute PD model mice (*n* = 3). (a) Immunohistochemistry of TH in midbrain SN and striatum (× 400); (b-c) average optical density analysis of TH levels in the substantia nigra and striatum. ⁣^∗^*p* < 0.05, ⁣^∗∗^*p* < 0.01 compared to the model group.

The TH immunoreactive substances in the SN and midbrain striatum were brownish-yellow under a light microscope, mostly distributed in the cytoplasm. The results showed that TH-positive cells had lighter brown-yellow staining in the cytoplasm and the number of cells was significantly reduced in the model group compared with the blank group. TH-positive cells in the CA group had significantly enhanced brown-yellow staining in the cytoplasm compared with the model group (Figures 3(a)). The average optical density of TH in the midbrain SN and striatum of the model group was significantly reduced (*p* < 0.01) compared to the control group. In the CA-150, 300, and 600 mg/kg groups, there was no significant increase in TH in the SN compared to the model group (*p* > 0.05) (Figure 3(b)). However, the average optical density of TH in the striatum of the CA group was significantly increased (*p* < 0.05, *p* < 0.01) (Figure 3(c)).

**Figure 4:**
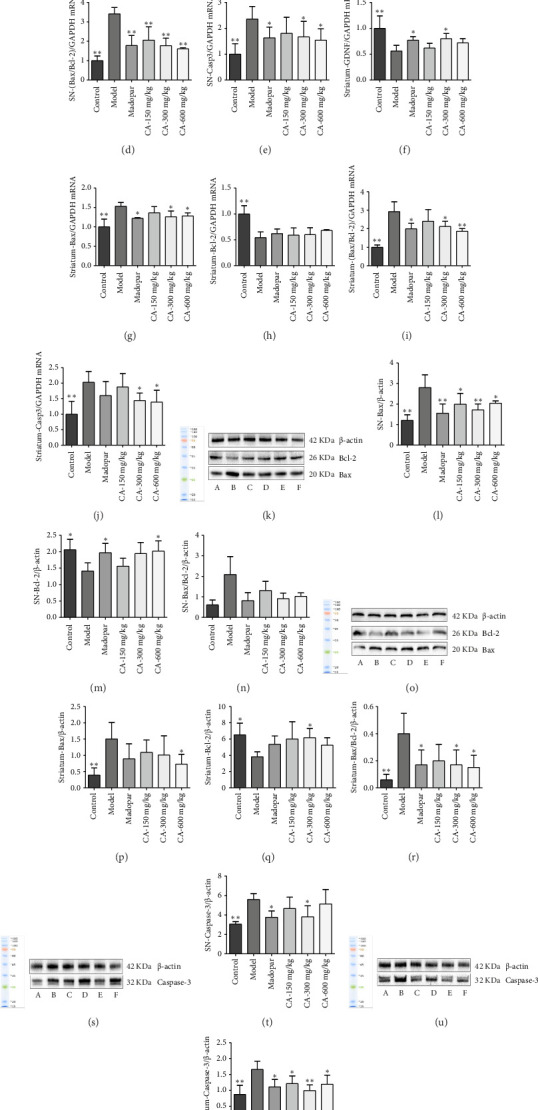
Effects of CA on mRNA and protein expression of GDNF, Bax, Bcl-2, and Casp3 in midbrain SN and striatum of MPTP-induced acute PD model mice (*n* = 3). (a–e) *GDNF*, *Bax*, *Bcl-2*, *Bax*/*Bcl-2*, and *Casp3* mRNA levels in the SN of different groups; (f–j) expression of *GDNF*, *Bax*, *Bcl-2*, *Bax/Bcl-2*, and *Casp3* mRNA in the striatum of different groups; (k) detection of Bax and Bcl-2 in the SN using Western blot; (l–n) analysis of the gray value of Bax and Bcl-2 in the SN; (o) detection of Bax and Bcl-2 protein expression in the striatum using Western blot; (p–r) analysis of the gray value of Bax and Bcl-2 in the striatum; (s) detection of Caspase3 in the SN using Western blot; (t) analysis of the gray value of Caspase3 in the SN; (u) detection of Caspase3 in the striatum using Western blot; (v) analysis of the gray value of Caspase3 in the striatum. ⁣^∗^*p* < 0.05, ⁣^∗∗^*p* < 0.01 compared to the model group.

The expression of *GDNF* mRNA in the midbrain SN and striatum was significantly decreased (*p* < 0.05) in the model group compared to the control group. However, the expression of *GDNF* mRNA significantly increased (*p* < 0.05) in the striatum of the CA-300 mg/kg group. There was no significant difference in *GDNF* mRNA of the midbrain SN. The expression levels of *Bax*, *Bax/Bcl-2*, *Casp3* mRNA, and protein content were significantly higher (*p* < 0.01) in the midbrain SN and striatum of the model group compared to the control group, while Bcl-2 mRNA and protein were significantly lower (*p* < 0.01). In the CA-150, 300, and 600 mg/kg groups, the mRNA and protein expression levels of *Bax*, *Bax/Bcl-2*, and *Casp3* were significantly decreased (*p* < 0.05) in the midbrain SN and striatum compared to the model group. The expression level of *Bcl-2* mRNA showed a nonsignificant increase, whereas the expression level of Bcl-2 protein was significantly elevated (*p* < 0.05), particularly in the CA-300 mg/kg group (Figure 4).

### 3.4. Augmentation of Neurotransmitter Content in Striatum by CA

**Figure 5:**
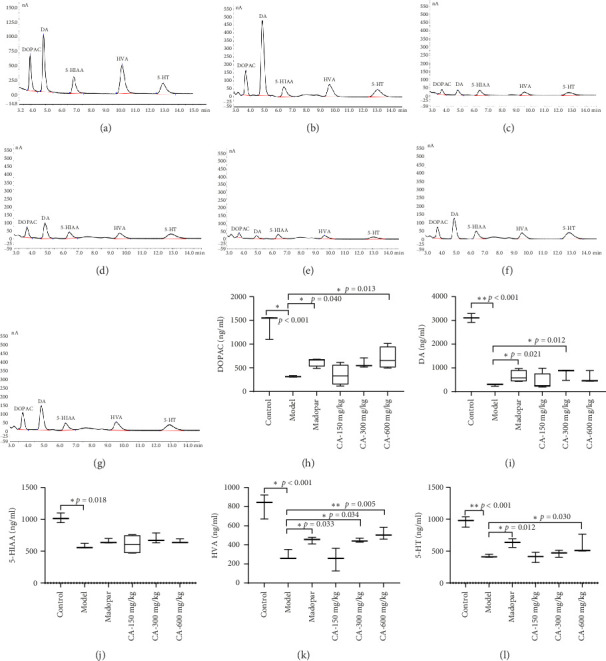
High-performance liquid chromatography of neurotransmitter standards and CA on neurotransmitters in the striatum of MPTP-induced PD mice (*n* = 5). (a) Chromatogram of five neurotransmitters in the standard; (b) chromatogram of five neurotransmitters in the control group; (c) chromatograms of five neurotransmitters in the model group; (d) chromatogram of five neurotransmitters in the Madopar group; (e) chromatogram of five neurotransmitters in the CA-150 mg/kg group; (f) chromatogram of five neurotransmitters in the CA-300 mg/kg group; (g) chromatogram of five neurotransmitters in the CA-600 mg/kg group; (h–l) quantification of the changes in neurotransmitters between different groups. ⁣^∗^*p* < 0.05, ⁣^∗∗^*p* < 0.01 compared to the model group.

Before the measurement, we first conducted a linear range using the standard sample of the DOPAC, DA, 5-HIAA, HVA, and 5-HT. The regression equation and correlation coefficient of the standard curve demonstrated a robust linear relationship, affirming the method's reliability (Table 2). Compared to the model group, the levels of DOPAC, HVA, and 5-HT in the striatum of mice in the CA-600 mg/kg group exhibited a significant increase. And the levels of DA in the striatum of mice in the CA-300 mg/kg group exhibited a significant increase. However, there was no notable increase in the level of 5-HIAA (Figure 5).

### 3.5. Inhibition of Glial Cell Activation by CA

**Figure 6:**
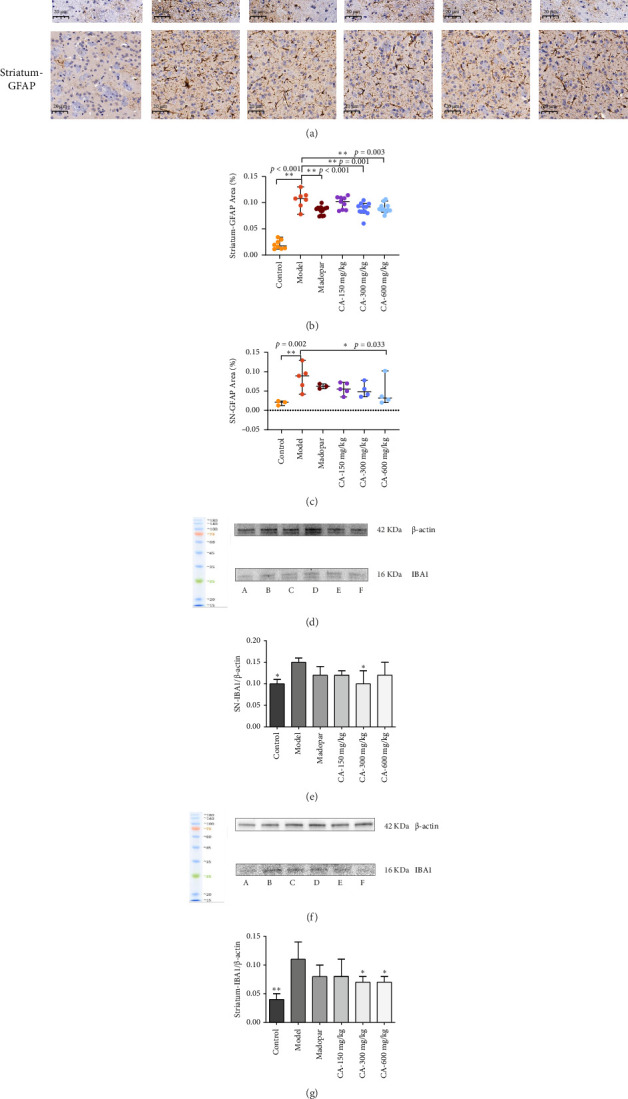
Effect of CA on the expression of IBA1 and GFAP protein in the substantia nigra (SN) and striatum of MPTP-induced acute PD model mice (*n* = 3). (a) Immunohistochemistry of GFAP in midbrain SN and striatum (× 400); (b-c) average optical density analysis of GFAP levels in the substantia nigra and striatum; (d) IBA1 in the substantia nigra using Western blot; (e) analysis of the gray value of IBA1 in the substantia nigra; (f) striatal IBA1 using Western blot; (g) analysis of the gray value of IBA1 in the striatum. ⁣^∗^*p* < 0.05, ⁣^∗∗^*p* < 0.01 compared to the model group.

The expression of GFAP-immunoreactive substances in the midbrain SN and striatum was brownish-yellow in color under a light microscope, mostly distributed in the cytoplasm, and the nuclei of the cells were stained blue by hematoxylin. The results showed that GFAP-positive cells in the model group had enhanced brownish-yellow staining in the cytoplasm. The number of cells was significantly reduced compared with the blank group. The brown-yellow staining in the cytoplasm of GFAP-positive cells in the CA group was significantly lighter compared with the model group (Figure 6(a)). The average optical density of GFAP in the SN and striatum of the model group significantly increased (*p* < 0.01) compared to the control group. In contrast, compared to the model group, the average optical density of GFAP in the SN CA-600 mg/kg group significantly reduced (*p* < 0.01), and significantly decreased (*p* < 0.01) in the striatum CA (300, 600 mg/kg) group (Figures 6(b) and 6(c)). Additionally, the expression level of IBA1 protein in the midbrain SN and striatum of the model group significantly increased compared to the control group (*p* < 0.01). Conversely, compared to the model group, the expression of IBA1 protein in the midbrain SN and striatum of mice in the CA-300 and 600 mg/kg groups significantly decreased (*p* < 0.05).

### 3.6. Attenuation of Inflammatory Factors and Reduction in Neuroinflammatory Response by CA

**Figure 7:**
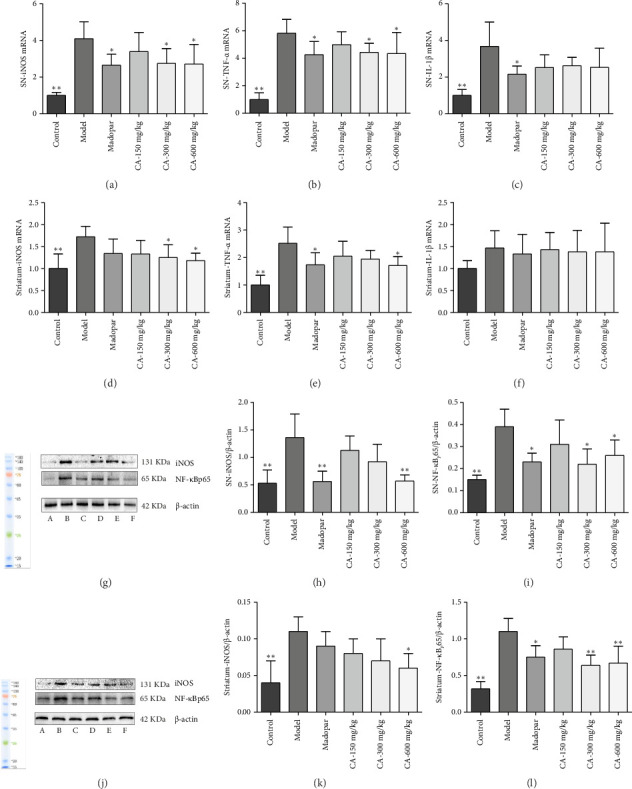
Effects of CA on gene and protein expression levels of inflammatory factors in the substantia nigra (SN) and striatum of MPTP-induced acute PD model mice (*n* = 3). (a–c) Expression of *iNOS*, *TNF-α*, and *IL-1β* mRNA in the substantia nigra; (d–f) mRNA expression of *iNOS*, *TNF-α*, and *IL-1β* in the striatum; (g) utilization of Western blot to detect iNOS and NF-*κ*Bp65 in the substantia nigra; (h–i) analysis of the gray values of iNOS and NF-*κ*Bp65 in the substantia nigra; (j) employing Western blot to detect iNOS and NF-*κ*Bp65 in the striatum; (k–l) analysis of the gray values of iNOS and NF-*κ*Bp65 in the striatum. ⁣^∗^*p* < 0.05, ⁣^∗∗^*p* < 0.01 compared to the model group.

The expression of inflammatory factors in the brain tissue of each group of mice was assessed via RT-qPCR and WB at the gene and protein levels, respectively. Mice in the CA-300 and 600 mg/kg groups displayed significantly lower levels of *iNOS*, *TNF-α*, and *IL-1β* mRNA in the midbrain SN compared to the model group (*p* < 0.05), along with diminished iNOS protein expression (*p* < 0.01). The expression level of NF-*κ*B p65 protein in the CA-300/600 mg/kg group significantly decreased (*p* < 0.05). Furthermore, the expression level of *iNOS* mRNA in the striatum of the CA-300 and 600 mg/kg groups significantly decreased (*p* < 0.05). The expression level of *TNF-α* mRNA in the striatum of the CA-300 mg/kg group significantly reduced (*p* < 0.05). However, there was no significant difference in the expression level of *IL-1β* mRNA in the CA group (*p* > 0.05). At the protein level, the expression level of iNOS protein in the striatum of the CA-600 mg/kg group significantly decreased (*p* < 0.05), and the expression level of NF-*κ*B p65 protein in the CA-300 and 600 mg/kg groups significantly decreased (*p* < 0.01) (Figure 7).

## 4. Discussion

In this study, we used MPTP to replicate the classic PD model [13]. After MPTP injection, the crossings, standing times, and staying time of the mice were significantly reduced, the climbing time was significantly prolonged, and the symptoms of obvious dyskinesia and impaired coordination behavior were observed. After CA treatment, the crossing grid, standing times, and staying time of the mice were significantly increased, and the climbing time was significantly shortened. MPTP modeling can make the behavioral changes of animals very similar to those of clinical PD patients [17], and this model is very beneficial for the study of PD. The decrease of DA nerve function in the SN–striatum pathway is a typical clinical symptom of PD patients. TH, as a rate-limiting enzyme in the synthesis of catecholamines such as DA, can be used as a marker of midbrain neurons [18]. In this study, it was found that MPTP injection would lead to a decrease in the expression of TH in the SN and striatum. After CA treatment, the expression of TH in the SN and striatum was significantly upregulated. It is indicated that CA can protect DA neurons by maintaining TH levels in the midbrain SN and striatum and significantly improving motor nerve damage in MPTP-induced acute PD mice.

During the pathogenesis of PD, neuroinflammation is activated mainly through brain-resident immune cells in the pathophysiology of PD [19]. GFAP and IBA1 are markers for triggering astrocytes and microglia [20]. Microglia, as intrinsic immune cells in the brain, are the first line of defense against brain injury and can participate in the basic physiological functions and pathological processes of the central nervous system. Under normal physiological conditions, microglia in the resting state mainly play the roles of immune defense, immune surveillance, and tissue repair. When nerve cells are damaged or subjected to inflammatory stimuli, microglia are activated and release several inflammatory mediators, such as TNF-α, IL-1β, iNOS, and so on. The continuous production and accumulation of these toxic mediators further induce the reactivation of microglia, and the activated microglia release inflammatory factors and neurotoxins. Therefore, the damaged DA neurons and activated microglia constitute a self-driven “vicious circle,” which plays a key role in the development of PD. Astrocytes are the most abundant glial cells in the CNS, which can protect neurons by releasing neurotrophic factors, producing antioxidant factors, and so on. MPTP modeling decreases the ability of astrocytes to remove α-syn from DAergic neurons, and the increase in reactive astrocytes exacerbates neurotoxicity by increasing the release of proinflammatory factors, which in turn exacerbates neuronal death. In addition, the interaction between microglia and astrocytes was found to be a key factor in regulating astrocyte function. Activated neuroinflammatory microglia induced astrocytes to switch to type A1, depriving them of their ability to promote neuronal survival, growth, synaptogenesis, and phagocytosis, and generating the ability to induce neuronal and oligodendrocyte death. The results of the present experiments showed that MPTP injection induced an increase in the average optical density of GFAP and an increase in the relative expression of IBA1 protein in the SN and striatum of the midbrain of mice, activating astrocytes and microglia.

TNF-α and other cytokines have the capacity to amplify the inflammatory response by activating the NF-*κ*B pathway. NF-*κ*B is prevalent in microglia, astrocytes, and neurons. Typically, NF-*κ*B binds to inactive I*κ*B. Upon external stimulation of resident immune cells in the brain (astrocytes and microglia), glial cells are activated, leading to the production of inflammatory cytokines such as TNF-α, IL-1β, and iNOS. Inflammatory cytokines phosphorylate and degrade I*κ*B kinase-beta (IKK-β), a specific subunit of I*κ*B. This action prompts NF-*κ*B to translocate from the cytoplasm to the nucleus, resulting in increased expression of inflammatory genes within cells. This process aids neuroglia in exerting their neurotoxic effects [21]. Animals treated with CA exhibited reduced levels of *iNOS*, *TNF-α*, and *IL-1β* mRNA and protein in the SN and striatum. This demonstrates that CA can diminish the release of inflammatory mediators by inhibiting the activation of glial cells, subsequently impeding the NF-*κ*B signaling pathway's activation. This reduction in neuroinflammatory response protects DA neurons.

The glial-derived neurotrophic factor (GDNF) plays a crucial role as a pro-survival factor for DA neurons in the SN–striatum pathway of PD. GDNF is the preferred neurotrophic factor for the neuroprotection treatment of PD [22, 23]. Studies have indicated that GDNF treatment can restore some clinical symptoms in Parkinsonian monkeys [24] and improve dyskinesia in PD patients [25]. Therefore, the decrease in GDNF expression in the SN of PD patients may directly contribute to the death of DA neurons and disease progression. In our experiment, the level of *GDNF* mRNA in the SN and striatum of PD model mice in the model group decreased. Following CA administration, there was an increase in the expression level of *GDNF* mRNA, suggesting that CA administration may alleviate and delay the motor symptoms of PD by upregulating the level of *GDNF* mRNA, providing a neuroprotective effect.

Apoptotic cell death significantly contributes to DA neurodegeneration [26]. Pro-apoptotic factors (Bax and Caspase-3) and anti-apoptotic markers (Bcl-2) belong to the Bcl-2 family and are pivotal controllers of the mitochondrial apoptotic cell death pathway [27]. Studies have demonstrated that MPTP triggers the apoptotic cell death process, leading to DA neuron death [28]. The ratio of Bax to Bcl-2 indicates the activation of the apoptotic mitochondrial cascade pathway, correlating with the incidence of PD. Our findings indicate that CA downregulated the expression of Casp3, Bax protein, and mRNA while upregulating the expression of Bcl-2, Bax/Bcl-2 protein, and mRNA in the SN and striatum of the mouse midbrain. CA exerts anti-apoptotic effects by increasing GDNF expression and inhibiting the activation of the apoptotic mitochondrial cascade pathway, ultimately protecting DA neurons.

## 5. Conclusion

In summary, CA not only ameliorated motor symptoms but also augmented neurotransmitter levels in the striatum of MPTP-induced acute PD model mice. The mechanism may be attributed to the following factors: Firstly, CA can mitigate the neuroinflammatory response and safeguard DA neurons by impeding the activation of astrocytes and microglia in the brains of MPTP-induced acute PD mice. Secondly, CA can restrain the activation of the apoptotic mitochondrial cascade pathway by upregulating the expression of GDNF, fostering the expression of Bcl-2, suppressing the expression of Bax and Casp3, and reducing the Bax/Bcl-2 ratio. This inhibition impedes the onset of apoptosis in DA neurons in the SN, thereby playing a neuroprotective role. Meanwhile, we also noticed that there were some limitations of the study. First, although the biological replicates required for gene and protein determination are at least three samples, we also know that the larger the sample size, the better the data's accuracy. Especially, some data didn't show a dose–effect relationship such as the behavior ability in the CA-300 and 600 mg/kg groups, which may be related to the sample size and individual differences. Therefore, it is necessary to increase the sample size to reduce individual differences and make the data more accurate. Second, *GAPDH* gene variants in the human genome induce pseudogene expression, especially in tumor and aging tissues [29]. We will further investigate whether *GAPDH* gene is suitable as internal reference for PD animal models.

## Figures and Tables

**Table 1:** Information for the primers used in this study.

Number	Gene name	Primers	Sequence 5′–3′	Product length (bp)
NM_010927.4	*iNOS* (mouse)	Forward	GTTCTCAGCCCAACAATACAAGA	127
		Reverse	GTGGACGGGTCGATGTCAC	

NM_001278601.1	*TNF-α* (mouse)	Forward	CTATGGCCCAGACCCTCACA	103
		Reverse	TTGAGATCCATGCCGTTGG	

NM_008361.4	*IL-1β* (mouse)	Forward	GCAACTGTTCCTGAACTCAACT	89
		Reverse	ATCTTTTGGGGTCCGTCAACT	

NM_001301332.1	*GDNF* (mouse)	Forward	GATCTCCAGGCAAGACCTCG	580
		Reverse	TTCAGGCATATTGGCGGCG	

NM_007527.4	*BAX* (mouse)	Forward	GTGAGCGGCTGCTTGTCT	68
		Reverse	GGTCCCGAAGTAGGAGAGGA	

NM_009741.5	*Bcl-2* (mouse)	Forward	GTACCTGAACCGGCATCTG	76
		Reverse	GGGGCCATATAGTTCCACAA	

NM_009810.3	*Casp3* (mouse)	Forward	GAGGCTGACTTCCTGTATGCTT	77
		Reverse	AACCACGACCCGTCCTTT	

NM_001289726.2	*GAPDH* (mouse)	Forward	AGGTCGGTGTGAACGGATTTG	123
		Reverse	TGTAGACCATGTAGTTGAGGTCA	

**Table 2:** Regression equation and correlation coefficient of standard substance.

Neurotransmitter	Linear regression equation	Correlation coefficient (*R*^2^)
DOPAC	*y* = 0.1962*x* − 0.1381	0.9996
DA	*y* = 0.3668*x* − 0.3713	0.9997
5-HIAA	*y* = 0.1963*x* − 0.5422	0.9997
5-HT	*y* = 0.2584*x* − 0.5263	0.9997
HVA	*y* = 0.3503*x* − 0.9277	0.9999

## Data Availability

Data will be made available from the first author and corresponding author upon request.

## Nomenclature

CA: Cinnamaldehyde
PD: Parkinson's disease
DBS: Deep brain stimulation
SN: Substantia nigra
DBS: Deep brain stimulation
MPTP: 1-Methyl-4-phenyl-1,2,3,6-tetrahydropyridine
TH: tyrosine hydroxylase
DA: Dopamine
5-HT: 5-hydroxytryptamine
DOPA: Dihydroxyphenylacetic acid
HVA: Homo vanillic acid
5-HIAA: 5-hydroxy indole acetic acid
